# Alkaline persulfate oxidation as an intermediate step for the development of a wet chemical oxidation interface for compound-specific *δ*^15^N analysis by LC-IRMS

**DOI:** 10.1007/s00216-025-05795-2

**Published:** 2025-02-22

**Authors:** Daniel Köster, Tobias Hesse, Felix Niemann, Maik A. Jochmann, Torsten C. Schmidt

**Affiliations:** 1 Instrumental Analytical Chemistry, University of Duisburg-Essen, Universitätsstr. 5, 45141 Essen, Germany; 2 University of Duisburg-Essen, Centre for Water and Environmental Research (ZWU) Universitätsstr. 5, 45141 Essen, Germany; 3 Probenahmedienst Feststoffe, Ressourcen- und Qualitätsmanagement, Landesamt Für Natur, Umwelt und Verbraucherschutz NRW, Wuhanstr. 6, 47051 Duisburg, Germany; 4 Institut Für Arbeitsschutz der Deutschen Gesetzlichen Unfallversicherung (IFA), Alte Heerstraße 111, 53757 Sankt Augustin, Germany

**Keywords:** LC-IRMS, Compound-specific isotope analysis, Peroxydisulfate, Alkaline oxidation

## Abstract

For the measurement of compound-specific isotope ratios by liquid chromatography isotope ratio mass spectrometry (LC-IRMS), complete mineralization of organic compounds to a single species of measurement gas is required so that isotopic fractionation can be minimized and corrected by identical treatment with standards. The established use of peroxydisulfate in an acidic environment has its limitations, especially when it comes to the complete oxidation of nitrogen-containing compounds with aromatic ring systems. Under acidic oxidation conditions, ammonium and nitrate were identified as the main nitrogen containing mineralization products of the oxidation of different model compounds. In contrast to the oxidation in an acidic environment, alkaline peroxydisulfate oxidation leads to nitrate as a final mineralization product. The concept of alkaline oxidation was transferred from large-scale batch experiments to a commercially available oxidation reactor used in LC-IRMS systems. The obtained nitrate recoveries indicate that alkaline oxidation could be a promising step towards the measurement of compound-specific nitrogen isotope ratios by LC-IMRS. In our work, we show that alkaline peroxydisulfate oxidation allows faster and more complete mineralization of nitrogen-containing compounds. For several model compounds, 63 to 100% of the initially present nitrogen was converted to nitrate within a reaction time of 43 s.

**Graphical Abstract:**

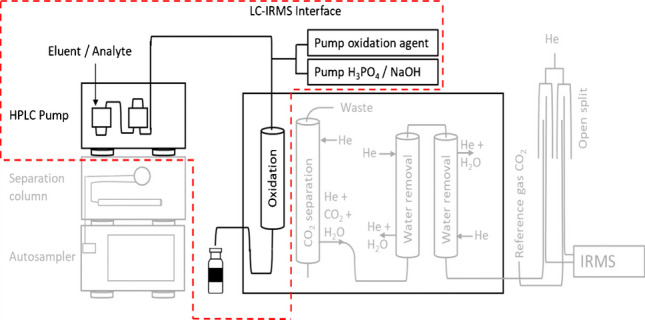

**Supplementary Information:**

The online version contains supplementary material available at 10.1007/s00216-025-05795-2.

## Introduction

Liquid chromatography coupled to isotope ratio mass spectrometry (LC-IRMS) is a technique that is nowadays routinely employed for the purpose of compound-specific isotope analysis (CSIA), through which carbon isotope ratios of individual compounds, such as sugars [1] and sweeteners [2], can be obtained. Analytes are separated by liquid chromatography and introduced into the LC-IRMS interface, where they are oxidized by heat-activated persulfate in a wet chemical oxidation reactor. The resulting CO_2_ is separated from the liquid mobile phase by membrane pervaporation and transferred to the IRMS for isotope ratio determination [3]. In contrast to gas chromatography isotope ratio mass spectrometry (GC-IRMS), LC-IRMS is suitable for the determination of isotope ratios of polar molecules that are not suitable for GC measurements without derivatization [4–7]. In GC-IRMS, stable isotope ratios of hydrogen, nitrogen, and carbon are routinely measured. LC-IRMS is currently limited to the measurement of *δ*^13^C isotope ratios with aqueous solvents and inorganic buffers. In the case of the measurement of dual-isotope data (*δ*^13^C and *δ*^15^N) of polar target molecules, such as the herbicides glyphosate and chloridazon, LC-IRMS is employed for the *δ*^13^C measurement in addition to the *δ*^15^N measurement following derivatization by GC-IRMS [8, 9]. For the assessment of transformation mechanisms of environmental contaminants, the application of dual element analysis has been shown to provide information that are not accessible by single isotope monitoring, e.g., *δ*^13^C analysis alone [10, 11]. Bulk nitrogen stable isotope analysis plays an important role in the study of food web structures in various ecosystems, as the heavier ^15^N isotope is enriched by 3 to 5‰ by each trophic level in the organism compared to the isotope ratios in the food sources [12, 13]. Especially in very complex trophic interactions such as host parasite systems, nitrogen stable isotope analysis could be applied to elucidate the feeding behavior [14, 15]. In contrast to bulk stable isotope measurements, CSIA could lead to valuable additional information in these fields by the monitoring of specific nitrogen containing marker compounds rather than the analysis of the whole nitrogen content by EA-IRMS systems. With the help of CSIA, it is possible to differentiate individual isotopic signature of essential and non-essential amino acids. It has been shown that essential amino acids such as phenylalanine show a small fractionation of < 0.5‰ per trophic transfer, whereas non-essential amino acids such as glutamic acid showed significant fraction in their nitrogen isotope ratio of about 6–8‰ [16]. Many published applications for *δ*^13^C analysis by LC-IRMS focusing on amino sugar analysis, the authenticity of caffeine containing drinks, the analysis of pharmaceuticals, and amino acids could benefit from the additional *δ*^15^N information which is not yet directly accessible by LC-IRMS alone [17–21].

Currently, the determination of *δ*^15^N isotopic signatures in addition to *δ*^13^C information of polar compounds that are not directly GC measurable is mostly performed on two parallel analytical platforms [8, 9]. Carbon isotope ratios can also be determined by GC after derivatization, but the introduction of carbon containing groups into the molecule will alter the isotopic signature of the analyte which has to be corrected afterwards [22]. In addition, the derivatization process has to be very well understood and controlled, so that incomplete derivatization cannot introduce isotopic shifts into the results. A combination of preparative LC separation and isotope measurement of nitrogen containing analytes such as amino acids by elemental analyzer IRMS (EA-IRMS) has been performed and showed increased precision of the *δ*^15^N results compared to GC-IRMS after derivatization [23]. The main drawback of this technique is that it cannot be combined into an online-system and that the experimental effort drastically increases with multi component LC separations. An online combination of LC separation and EA-IRMS measurement has been developed by Federherr et al., which combines LC with high temperature catalytic conversion of the analytes for *δ*^15^N measurements [24, 25]. The system has successfully been tested for the measurement of compound-specific carbon and nitrogen isotope ratios, but problems due to the introduction of large amounts of liquid mobile phase into the EA oxidation reactor and deactivation of the catalyst by mobile phase additives have restricted the further application so far [26]. To overcome these drawbacks, an instrumental solution based on the already well-established *δ*^13^C LC-IRMS interface architecture commercialized by Thermo Fisher and Elementar Analysensysteme (IsoLink™ and Liquiface™, respectively) using online wet chemical oxidation and separation of the analyte gas by a membrane system would be preferable.

The oxidation of nitrogen containing analytes in the LC-IRMS system has been shown to require special optimization of the reaction conditions, as especially nitrogen containing heterocyclic ring systems are prone to reduced oxidation efficiency in the interface [20, 27, 28]. Nevertheless, under standard LC-IRMS measurement conditions, nitrogen containing oxidation products of the wet chemical oxidation cannot be monitored directly. For the analysis of the nitrogen containing reaction products, ion chromatography methods were developed that are able to determine nitrate, nitrite, and ammonium in the presence of large excess of persulfate, sulfate, and potassium or sodium present in the oxidation reactor of the LC-IRMS interface [28].

In a recent study, we used a modified commercially available LC-IRMS interface that enables automated measurement of δ^15^N signatures from nitrate by online reduction of nitrate in two consecutive steps. First, vanadium(III)-chloride was used as a reducing agent to convert NO_3_^−^ to N_x_O_y_ under acidic conditions. The mix of nitrogen oxides is then transferred trough a membrane into a helium stream and reduced to nitrogen (N_2_) analysis gas via a hot copper reactor. To adapt this method for the nitrogen isotope measurement of organic compounds, different adaptations are necessary [29].

Outside the LC-IRMS context, the oxidation products formed by the reaction of nitrogen-containing species with heat-activated persulfate were evaluated for the determination of total nitrogen analysis in water samples [30]. Oxidation was performed in a batch process in 50-mL glass vessels autoclaved at 100–110 °C for more than 30 min. The determination of specific oxidation products and a recovery calculation based on the amount of initially introduced nitrogen compared to the measured species after oxidation were performed only at the end of the batch oxidation process [30, 31]. As the oxidation reaction in the IsoLink™ LC-IRMS interface has to take place in a much shorter time interval of < 1 min due to the flow conditions and dimensions of the oxidation reactor, the intermediate species formed during the initial phase of the oxidation reaction are of great importance in this context. The results obtained from the batch experiments could be used to identify suitable reaction conditions for application in the development of an on-line oxidation process for compound-specific *δ*^15^N isotope analysis.

Here, a direct comparison should be made between the oxidation of model compounds under acidic and initially alkaline oxidation conditions. Speciation of the nitrogen-containing reaction products over the course of the oxidation reaction should provide insight into the primary and secondary oxidation reactions taking place. The results obtained from the alkaline batch oxidation will be transferred to the LC-IRMS system to evaluate the applicability of the alkaline oxidation conditions for further steps in the development of a compound-specific* δ*^15^N LC-IRMS setup.

## Materials and methods

### Chemicals and reagents

For the batch reactor mineralization experiment, glycine (ReagentPlus, ≥ 99%, Sigma-Aldrich, Steinheim, Germany) and caffeine (p.a. 99.9% Sigma-Aldrich, Steinheim, Germany) were used. For mineralization experiments in the LC-IMRS interface, the aforementioned compounds as well as potassium nitrate (Fluka Chemika, > 99.0%, Seelze, Germany), ammonium sulfate (ReagentPlus, ≥ 99%, Sigma-Aldrich, Steinheim, Germany), urea (puris p.a., ≥ 99%, Sigma-Aldrich, Steinheim, Germany), and acetonitrile (HPLC gradient grade, Fisher Scientific, Loughborough, UK) were used. The calibration for the TOC analysis was performed with a 1000 mg L^−1^ TOC reference standard (Sigma-Aldrich, Steinheim, Germany). For the oxidation reactions, potassium peroxydisulfate (puriss. p.a., > 99%, Sigma-Aldrich, Steinheim, Germany) and sodium peroxydisulfate (99%, Riedel-de Haën, Seelze, Germany) were employed. Mobile phases for the IC systems were prepared from ultrapure water obtained from an ELGA Purelab Ultra system (Veolia Water Technologies, Ratingen, Germany). A 1 M nitric acid solution from Fluka Analytical (Seelze, Germany) was used for the mobile phase of the cation chromatography system. For the anion chromatography, the mobile phase was prepared from sodium carbonate and sodium bicarbonate (both puriss. p.a.) obtained from Riedel-de Haёn (Seelze, Germany) and acetonitrile (HPLC gradient grade) from Fisher Scientific (Loughborough, UK) [32].

### Mineralization of model compounds in a batch reactor

**Fig. 1:**
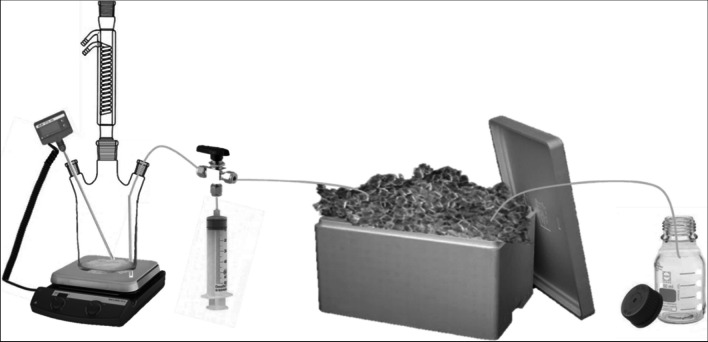
Setup for mineralization experiments: triple-necked flask with reflux condenser, automatic temperature control, manual three-way valve, and crushed ice cooling system

Mineralization of the analytes was performed in an experimental batch reactor setup consisting of a triple-necked glass flask, heated by a heating plate with automatic temperature control via a thermometer that was immersed in the reaction vessel. To avoid evaporation of water during the experiment, the flask was equipped with a reflux cooler. The third neck was used to withdraw the samples from the reactor. A stainless-steel capillary was connected to a three-port valve, leading to a 50-mL syringe and a cooling loop made of stainless-steel capillary immersed in crushed ice. With the help of a syringe, samples were withdrawn from the reactor and transported through the cooling loop after changing the position of the three-port valve. The reaction vessel was constantly agitated using a magnetic stir bar [32]. A schematic view of the setup also used for the mineralization experiments at neutral pH conditions is presented in Fig. 1.

Initially, the glass flask contained 1490 mL of water adjusted to the desired pH level by the addition of sulfuric acid or potassium hydroxide. After the reaction temperature of 96–98 °C was reached in the reactor, 10 mL of stock solution of the analyte was pipetted to obtain an initial carbon mass concentration of 50 mg L^−1^ (C). The first sample at *t*_0_ was withdrawn without the addition of K_2_S_2_O_8_. After the collection of the first sample, solid K_2_S_2_O_8_ and 50 mL water were added to obtain a volume of 1.5 L with a K_2_S_2_O_8_ concentration of 30 mmol L^−1^. At each subsequent time point, 50 mL samples were removed from the system without volume replacement. Of the 50 mL sample, 10 mL was discarded and used to purge the cooling loop, and the following 40 mL was collected for further analysis. From the 40 mL obtained, 10 mL aliquots were separated for cation and anion chromatography. For ion chromatography, 1 mL of methanol was added to each sample aliquot. The pH of the subsamples used for cation chromatography was adjusted by the addition of 0.5 mL of 1 M HCl for the alkaline oxidation experiments. The remaining 20 mL of the sample was used for the TOC measurement, and in the case of the alkaline oxidation conditions, 2 mL of 1 M HCl was added to the subsample for NPOC analysis. Individual samples were stored on ice until analysis. TOC analysis as well as pH was measurement for each time point (Metrohm 827 pH lab, Metrohm, Herisau, Switzerland) [32].

### LC-IRMS interface mineralization experiments

**Fig. 2:**
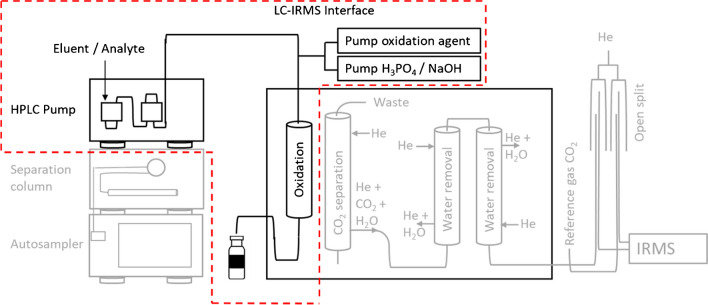
Modification of the LC-IRMS interface for the mineralization experiments

For the mineralization of analytes in the LC-IRMS system, the system was modified so that a constant stream of analyte solution at different concentrations was delivered directly to the oxidation reactor in the LC-IRMS interface (LC-IsoLink™, Thermo Fisher Scientific, Bremen, Germany) by a Rheos Allegro HPLC pump (Flux instruments AG, Basel, Switzerland) [32]. The setup of the system compared to the regular LC-IRMS system is shown in Fig. 2.

Sodium peroxydisulfate (100 g L^−1^ or 200 g L^−1^) and sodium hydroxide (1.25 mol L^−1^) were added to the interface at a flow rate of 50 µL min^−1^ or 25 µL min^−1^, respectively, depending on the specific experimental conditions. Oxidation in the LC-IRMS interface flow reactor was performed at 100 °C. After the oxidation reactor, the connection to the gas separation unit was replaced by a peek capillary leading to an ice-cooled sample vessel outside the interface. The analyte solution and the oxidation reagent and NaOH solution were continuously introduced into the instrument for at least 30 min to equilibrate and purge the system between different analytes or flow conditions. After equilibration, approximately 20 mL of sample was collected. Nitrate measurement by IC was performed immediately after sample collection [32].

### Measurement of residual organic carbon as NPOC

Measurement of non-purgeable organic carbon (NPOC) was performed using a TOC-L total organic carbon analyzer (Shimadzu, Duisburg, Germany). For the determination of NPOC, 20 mL of the sample from each time point of the mineralization experiment was transferred to open glass vials for the TOC-L autosampler (Shimadzu, Duisburg, Germany). The samples were acidified with 1 M HCl and purged with synthetic air (80 mL min^−1^) inside the needle body of the autosampler unit to remove dissolved inorganic carbon. After 3 min of purging, 50 µL of the sample was injected into an oxidation tube filled with platinum-coated ceramic particles at 720 °C. The CO_2_ produced during oxidation was measured by infrared spectroscopy. Calibration was performed with dilutions prepared from a certified TOC reference standard (Sigma Aldrich, 1000 ± 10 mg L^−1^ TOC) [32].

### Measurement of nitrate, nitrite, and ammonium by ion chromatography

Two distinct IC systems were employed for the quantification of nitrate, nitrite, and ammonium. Both comprised a Metrohm 883 Basic IC coupled with a Metrohm 863 Compact autosampler (both manufactured by Metrohm, Herisau, Switzerland). For the analysis of the anions, a Metrosep A Supp 4 (250/4.0) column (Metrohm, Herisau, Switzerland) was employed, with an eluent consisting of 1.8 mmol L^−1^ Na_2_CO_3_ and 1.7 mmol L^−1^ NaHCO_3_ in a 30/70 vol.-% acetonitrile/water mixture. An injection volume of 10 µL was employed, with a flow rate of 1 mL min^−1^ utilized for the anion separation. An ion suppression module (MSM, Metrohm, Herisau, Switzerland) was employed for anion chromatography with the objective of reducing the background conductivity of the eluent and enhancing sensitivity. For the separation of the cations, a Metrosep C4 (150/4.0) column (Metrohm, Herisau, Switzerland) with an eluent consisting of 4 mmol L^−1^ HNO_3_ in water at a flow rate of 0.9 mL min⁻^1^ was employed. The injection volume was 4 µL for the cation separation. Detection on both systems was performed with a conductivity detector. Calibration was conducted by a dilution of stock solutions prepared from NH_4_Cl and NaNO_3_ in the presence of 30 mmol L^−1^ PDS to obtain a comparable matrix as in the real samples from the mineralization experiments [32].

## Results and discussion

### Influence of pH on the oxidation of reference substances in a batch reactor system

**Fig. 3:**
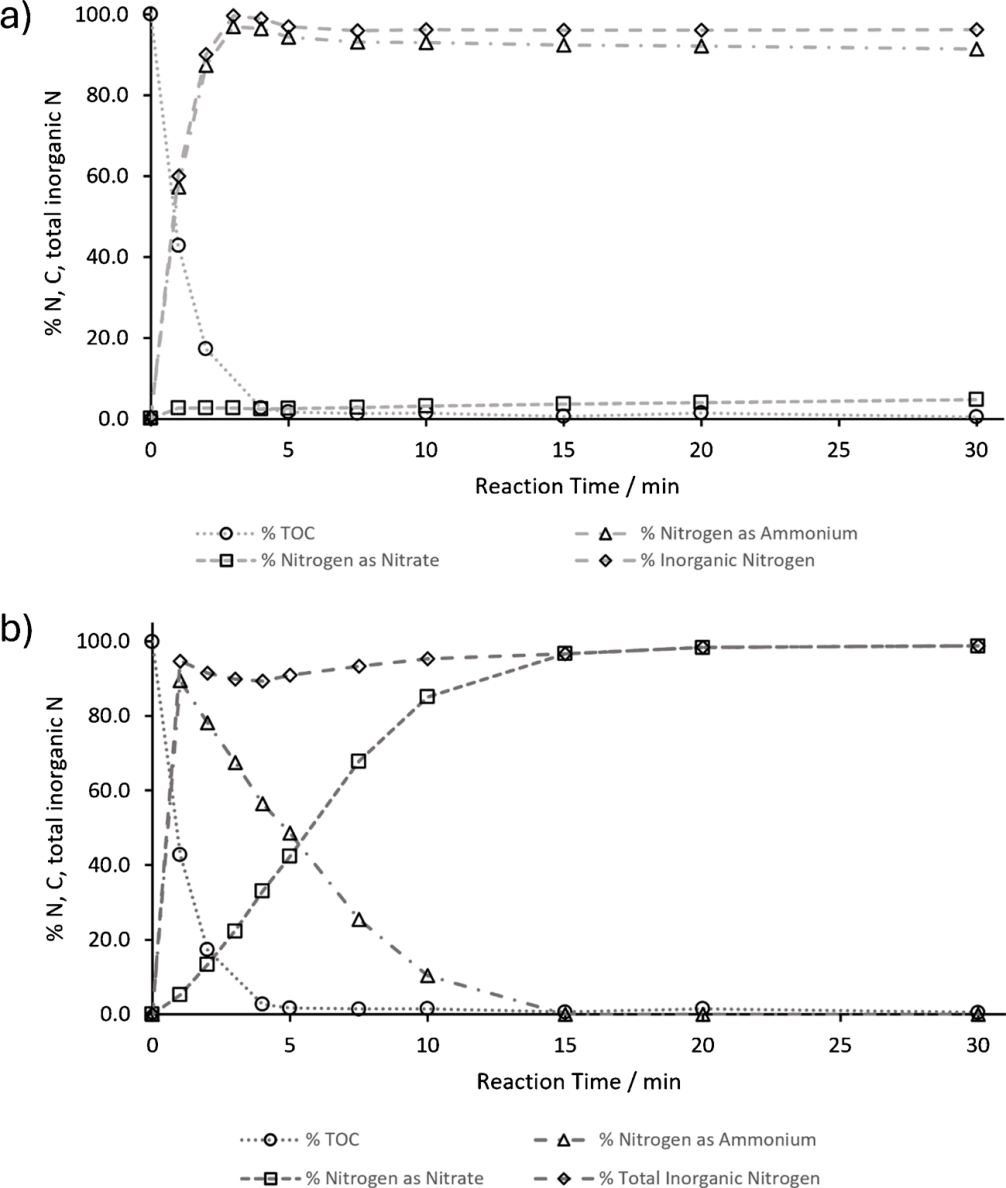
**a** Oxidation of glycine by heat-activated persulfate at an initial pH of 2 adjusted by sulfuric acid. **b** Oxidation of glycine by thermal-activated persulfate at an initial pH of 12 adjusted by potassium hydroxide

To investigate the influence of the initial pH in the oxidation of nitrogen containing organic molecules by thermal-activated persulfate, batch rector experiments with two reference substances (glycine and caffeine) were conducted. An initial system pH was chosen at pH 2 for the oxidation at acidic conditions and pH 12 for alkaline oxidation experiments. Total oxidation of the analyte was monitored by the amount of total organic carbon present at each time point during oxidation. Dissolved inorganic carbon as an oxidation product during alkaline oxidation was eliminated during TOC analysis by acidification and sparging of the sample. The inorganic nitrogen species nitrite, nitrate, and ammonium were monitored by IC measurements at each time point. For glycine, the results obtained for oxidation under acidic conditions are shown in Fig. 3a.

A comparison of the results obtained under the same experimental conditions except for the adjustment of the initial pH in the system to pH 12 by potassium hydroxide is presented in Fig. 3b.

**Fig. 4:**
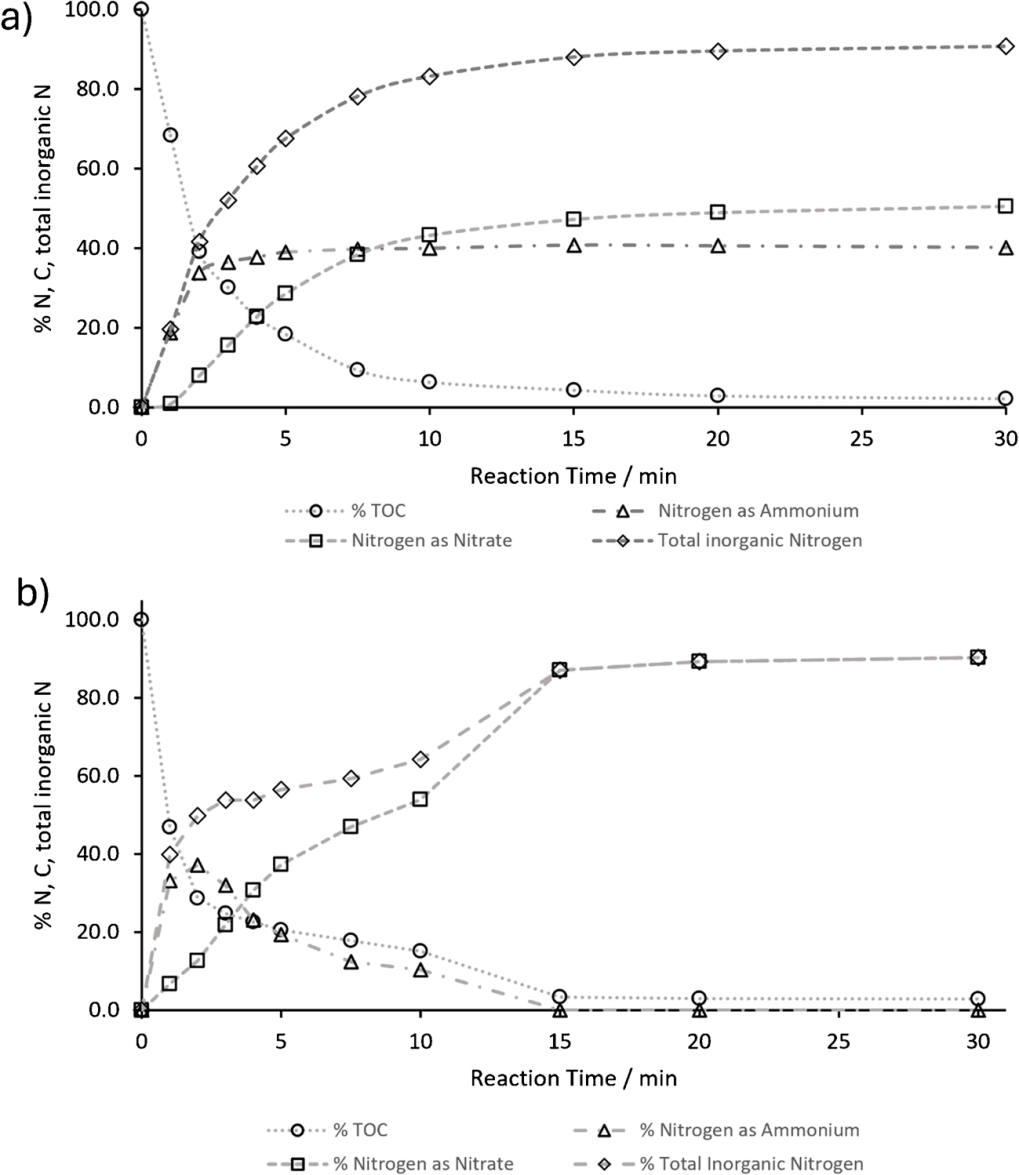
**a** Oxidation of caffeine by heat-activated persulfate at an initial pH of 2 adjusted by sulfuric acid. **b** Oxidation of caffeine by heat-activated persulfate at an initial pH of 12 adjusted by potassium hydroxide

In the initial stages of the experiment, under conditions of low pH (Fig. 3a), glycine is rapidly converted to CO₂ and ammonium, with the conversion occurring within the first 3 min. The oxidation of ammonium to nitrate is comparatively slower, with only 4.8% of the nitrogen present in the form of nitrate after 30 min of oxidation. In contrast to the acidic conditions, the alkaline conditions (pH 12) result in a faster conversion of the nitrogen present in the organic analyte to ammonium, with 90% of the nitrogen converted within the first minute of the reaction. Furthermore, the oxidation from ammonium to nitrate is much faster than under acidic conditions. Within the initial 15 min, 97% of the nitrogen is oxidized to nitrate. After 30 min of reaction time, 99% of the initially present organic nitrogen can be recovered as nitrate. The results from the oxidation of caffeine under acidic and alkaline conditions are shown in Fig. 4a and b**,** respectively.

In comparison to glycine, the mineralization of caffeine by heat-activated persulfate is overall slower, as indicated by the decrease of the organic carbon in the system. The nitrogen atoms in caffeine are bound in carbon ring systems and not in a freely accessible amino group, in contrast to glycine. For caffeine, it takes 15 min for the total organic carbon (TOC) to drop below 5% of the initially introduced carbon. Even within 30 min of oxidation, 2–3% of the carbon is not oxidized to CO₂ and remains in the system in a non-purgeable carbon form. These results are consistent with the observations from previous batch experiments [28] and mineralization experiments [32] and underline that analytes such as caffeine with C = N bond systems are more resistant to oxidation by heat-activated persulfate than analytes with single C-N bonds such as the amino group of glycine [27, 32].

In the presence of acid, the reaction of heat-activated persulfate and caffeine results in the formation of a mixture comprising 51% nitrate and 40% ammonium after 30 min. The remaining nitrogen present in the analyte is either bound in the residual organic fraction or present in an inorganic form that is not detected by the current IC method. Nitrite was monitored in all of the measurements but was never present in quantifiable amounts in the batch reactor samples, with a concentration below 0.5 mg L^−1^. A comparison of the formation of nitrate and ammonium between the initially alkaline and acidic reaction of heat-activated persulfate with caffeine reveals that parallel formation of both species can be observed in the first 5 min of the reaction. In contrast to the experiment at low pH, in the alkaline system, ammonium is oxidized to nitrate in parallel with the mineralization of caffeine. Following a 15-min period, the initially produced ammonium was almost completely oxidized to nitrate, whereas in the low pH system, a mixture of 41% ammonium and 47% nitrate was obtained. These results indicate that alkaline oxidation offers significant advantages for the application as an oxidation reaction in the LC-IRMS setup.

**Fig. 5:**
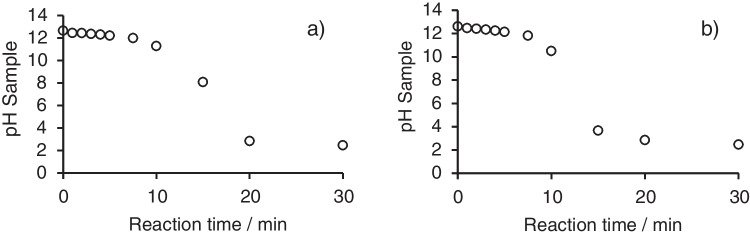
Change of the pH in the system during the batch oxidation under initial alkaline conditions: **a** oxidation of glycine and **b** oxidation of caffeine

In addition to the measurements of inorganic nitrogen species and TOC, the pH change in the reactor system was measured for the alkaline oxidation of glycine and caffeine. The observed drop of the pH value in the system during the oxidation of the respective analyte is shown in Fig. 5.

For both analytes, a notable decline in pH was observed in the reactor following a 10-min reaction period. The observed pH change can be attributed to the hydrolysis of persulfate at elevated temperatures in alkaline solutions, in accordance with the reaction mechanism [33].

$${S}_{2}{{O}_{8}}^{2-}+{H}_{2}O\ {\to }\ 2S{{O}_{4}}^{2-}+\frac{1}{2}{O}_{2}+2{H}^{+}$$

An application for the shift of the pH in a comparable batch measurement system for the parallel determination of total nitrogen (as nitrate) and phosphorous (as phosphate) has been published by De Borba et al. in 2014 [31]. Depending on the initial ratio of persulfate to hydroxide in the system, final pH levels of approximately 2 (persulfate to hydroxide ratio of 2:1 and 1:1) or pH 11 (persulfate to hydroxide ratio of 1:2) could be observed. In contrast to the application of De Borba et al., where a shift of the pH from alkaline to acidic was desired due to benefits for the phosphate measurement, in the context of an oxidation setup for the LC-IRMS application, the initial ratio of persulfate to hydroxide must be chosen in a way that the conditions during the oxidation remain alkaline to favor the oxidation of ammonium to nitrate. As indicated by Fig. 5, the pH in the batch system stayed above pH 10 in the first 10 min of the oxidation reaction, considering the relatively short reaction times currently possible in the LC-IRMS interface reactor (approx. 17–43 s); the conditions should be applicable for the fast oxidation of ammonium to nitrate in the interface setup.

### Application of the alkaline oxidation conditions in the IsoLink™ LC-IRMS interface

For the oxidation reaction in the flow-through reactor of the LC-IRMS interface, commonly used combinations of LC effluent entering the interface, as well as standard flow rates and concentrations of oxidation reagent (100 g L^−1^ Na_2_S_2_O_8_) and NaOH solution (50 g L^−1^) were used. The resulting pH values measured after the oxidation reaction at different flow rate from the HPLC (and thereby different NaOH concentration) as well as at different temperatures of the oxidation reactor and in combination with an infusion of organic analyte (glycine, 50 mg L^−1^ (C)) are shown in Table S1in the supporting information.

The pH conditions in the effluent of the interface did not drop below pH 12 for all evaluated combinations of flow rates from 200 µL min^−1^ (lower than commonly used in LC-IRMS applications) to a total flow of 700 µL min^−1^ which is the maximal flow allowed for the membrane separation unit in the LC-IRMS interface [34]. From the comparison of the pH values obtained at a temperature of 30 °C of the oxidation oven compared to an oxidation at 100 °C, only a relatively low drop of the pH could be observed for all flow rates. The addition of glycine as an exemplary analyte did also not lead to a significant change of the pH.

In the further evaluation of the oxidation under alkaline conditions in the LC-IRMS interface, glycine was used as a reference compound, as the batch experiments indicated a fast oxidation of the organic carbon and almost quantitative conversion of the initially present nitrogen to nitrate if the reaction time was long enough for the complete oxidation from ammonium to nitrate. In Table 1, the results for the oxidation of glycine at a set of different HPLC flow conditions are shown. In part (a), the oxidation agent concentration was 100 g L^−1^ Na_2_S_2_O_8_, whereas for the results shown in part (b), the oxidation agent concentration was increased to 200 g L^−1^. For each set of flows and oxidation agent concentrations, the nitrogen recovery as NO_3_ in relation to the initially present organic nitrogen is shown. For the interpretation of the results, the interaction of the changed parameters in the experimental setup has to be taken into account. With an increase of the analyte flows into the system, three parameters are simultaneously changed. With higher analyte flows, the analyte concentration in the oxidation reactor is increased (ratio of analyte flow to total flow); at the same time, the concentration of the oxidation agent is decreased (lower ratio of oxidation agent flow to HPLC flow), and the residence time of the mixture in the oxidation reactor is decreased (higher overall flow). Thereby, an increased analyte flow negatively affects the oxidation efficiency in three ways.

**Table 1:** Nitrogen recoveries as nitrate after the alkaline glycine oxidation in the IsoLink™ LC-IRMS interface reactor. (a) Oxidation agent concentration of 100 g L^−1^ Na_2_S_2_O_8_. (b) Oxidation agent concentration of 200 g L^−1^ Na_2_S_2_O_8_

a)								
Flows (µL min^−1^)				c_0_ (Glycine)	c (NO_3_^−^ after oxidation)	Recovery N	c (Na_2_S_2_O_8_)	Reaction time
Analyte flow	NaOH (50 g L^−1^)	Na_2_S_2_O_8_ (100 g L^−1^)	Total flow	(mg L^−1^ (N))	(mg L^−1^ (N))	%	(g L^−1^)	(s)
600	50	50	700	25.0	1.9	7.5	7.1	17
500	50	50	600	24.3	2.2	8.9	8.3	20
400	50	50	500	23.3	3.4	14.5	10.0	24
300	50	50	400	21.9	8.1	37.1	12.5	29
200	50	50	300	19.4	14.9	76.6	16.7	39
b)								
Flows (µL min^−1^)				c_0_ (Glycine)	c (NO_3_^−^ after oxidation)	Recovery N	c (Na_2_S_2_O_8_)	Reaction time
Analyte flow	NaOH (50 g L^−1^)	Na_2_S_2_O_8_ (200 g L^−1^)	Total flow	(mg L^−1^ (N))	(mg L^−1^ (N))	%	(g L^−1^)	(s)
600	50	50	700	25.0	5.1	20.6	14.3	17
500	50	50	600	24.3	7.9	32.4	16.7	20
400	50	50	500	23.3	12.6	54.1	20.0	24
300	50	50	400	21.9	17.5	80.1	25.0	29
200	50	50	300	19.4	18.6	95.5	33.3	39

From the results presented in Table 1 (part a), an increase in the nitrate recovery from 7.5 to 77% could be observed when the flows of the glycine solution were decreased from 600 to 200 µL min^−1^. At the same time, as a result of the overall lower system flow, the reaction time increased from 17 s in the oxidation reactor to 39 s. In comparison, in Table 1 (part b), the overall concentration of oxidation agent in the system was increased from 100 g L^−1^ Na_2_S_2_O_8_ to 200 g L^−1^ Na_2_S_2_O_8_, whereas the remaining flow conditions were kept constant. On the one hand, an increased oxidation agent concentration leads to an overall improved nitrogen recovery. On the other hand, a 100% increased oxidation agent concentration did not have the same effect on the nitrogen recovery as a 100% increase in the reaction time.

For low system flows (300 µL min^−1^) and high oxidation agent concentrations (200 g L^−1^ Na_2_S_2_O_8_), nitrogen recoveries above 95% can be obtained with the standard LC-IMRS interface. Lower flows are not applicable in the context of LC-IRMS, as low sensitivity of the instrument requires high sample amounts to be introduced which cannot be efficiently separated on columns designed for flows in the region below 100 µL min^−1^. The amount of oxidation agent added in the interface is finally limited by the solubility of Na_2_S_2_O_8_, which is 547.6 g L^−1^ at 20 °C [35]. The main drawback of increased oxidation agent concentrations is that the amount of oxygen produced during the oxidation is simultaneously increased. For the conventional LC-IMRS setup, this leads to a fast degradation of the EI filament and decreased precision of the isotope measurements, but excess oxygen can be removed by the use of an additional copper reduction system for the interface which also needs to be applied if gas phase reduction of nitrogen species to N_2_ is required in the transformation of nitrate to the IRMS measurement gas [36]. For the infusion experiments, potassium nitrate, ammonium sulfate, caffeine, glycine, urea, and acetonitrile were oxidized in the LC-IMRS interface under alkaline conditions (Table 2).

**Table 2:** Oxidation experiments by infusion at alkaline pH in the LC-IRMS interface reactor. Initial analyte concentrations were kept constant at 50 mg L^−1^ (N)

a)								
Flow (µL min^−1^)				c_0_ KNO_3_	c (NO_3_^−^ after oxidation)	Recovery N	c (Na_2_S_2_O_8_)	Reaction time
Analyte flow	NaOH (50 mg L^−1^)	Na_2_S_2_O_8_ (200 g L^−1^)	Total flow	(mg L^−1^ (N))	(mg L^−1^ (N))	%	(g L^−1^)	(s)
200	25	50	275	36.4	36.4	101.2	36.4	43
300	25	50	375	40.0	40.0	102.1	26.7	31
b)								
Flow (µL min^−1^)				c_0_ (NH_4_)_2_SO_4_	c (NO_3_^−^ after oxidation)	Recovery N	c (Na_2_S_2_O_8_)	Reaction time
Analyte flow	NaOH (50 mg L^−1^)	Na_2_S_2_O_8_ (200 g L^−1^)	Total flow	(mg L^−1^ (N))	(mg L^−1^ (N))	**%**	(g L^−1^)	(s)
200	25	50	275	36.4	36.3	99.8	36.4	43
300	25	50	375	40.0	40.1	100.2	26.7	31
c)								
Flow (µL min^−1^)				c_0_ (Caffeine)	c (NO_3_^−^ after oxidation)	Recovery N	c (Na_2_S_2_O_8_)	Reaction time
Analyte flow	NaOH (50 mg L^−1^)	Na_2_S_2_O_8_ (200 g L^−1^)	Total flow	(mg L^−1^ (N))	(mg L^−1^ (N))	**%**	(g L^−1^)	(s)
200	25	50	275	36.4	24.0	66.1	36.4	43
300	25	50	375	40.0	14.5	36.2	26.7	31
d)								
Flow (µL min^−1^)				c_0_ Glycine	c (NO_3_^−^ after oxidation)	Recovery N	c (Na_2_S_2_O_8_)	Reaction time
Analyte flow	NaOH (50 mg L^−1^)	Na_2_S_2_O_8_ (200 g L^−1^)	Total flow	(mg L^−1^ (N))	(mg L^−1^ (N))	**%**	(g L^−1^)	(s)
200	25	50	275	36.4	31.1	85.5	36.4	43
300	25	50	375	40.0	20.1	50.2	26.7	31
e)								
Flow (µL min^−1^)				c_0_ Urea	c (NO_3_^−^ after oxidation)	Recovery N	c (Na_2_S_2_O_8_)	Reaction time
Analyte flow	NaOH (50 mg L^−1^)	Na_2_S_2_O_8_ (200 g L^−1^)	Total flow	(mg L^−1^ (N))	(mg L^−1^ (N))	%	(g L^−1^)	(s)
200	25	50	275	36.4	28.2	77.4	36.4	43
f)								
Flow (µL min^−1^)				c_0_ Acetonitrile	c (NO_3_^−^ after oxidation)	Recovery N	c (Na_2_S_2_O_8_)	Reaction time
Analyte flow	NaOH (50 mg L^−1^)	Na_2_S_2_O_8_ (200 g L^−1^)	Total flow	(mg L^−1^ (N))	(mg L^−1^ (N))	%	(g L^−1^)	(s)
200	25	50	275	36.4	22.7	62.5	36.4	43

From the initially introduced analyte concentration, the maximal achievable nitrate recoveries (100% N oxidation to nitrate) were calculated. After taking the dilution of the analytes in the interface by the NaOH solution and the oxidation agent into account, nitrate recoveries were determined for each compound and set of reaction conditions. The infusion of nitrate solution (Table 2, part a)) was used as a control experiment to prove the precision of the dilution by the three independent pumps in the system which could have an effect on the results. For nitrate, recoveries of 101% and 102% were determined at system flows of 275 µL and 375 µL, respectively. Therefore, it can be concluded that the pump system was optimized to produce exact flows that allow a reliable calculation of the recoveries after oxidation. In addition, introduction of excess nitrate by the reagent solutions could be excluded. In the next step, an ammonium sulfate solution was infused into the interface (Table 2, part b)). At the given flow conditions, a complete oxidation of the nitrogen introduced as ammonium to nitrate could be achieved. This indicates that for more complex molecules, the initial oxidation of the analyte molecule is the critical step in the oxidation rather than the transformation of ammonium to nitrate. For caffeine (Table 3, part c)), which is known to be comparably recalcitrant to oxidation by heat-activated persulfate, nitrate recoveries of 66% and 36% for system flows of 275 µL min^−1^ and 375 µL min^−1^ could be obtained. For glycine, which is oxidized faster as indicated by the batch experiments, recoveries of 86% and 50% for residence times of 43 s and 31 s, respectively, were determined. This is comparable to the results presented in Fig. 1b**)** for system flows in the interface of 300 µL min^−1^ and 400 µL min^−1^ although the flow of NaOH solution was decreased by 50%. For urea and acetonitrile (Table 2, parts e and f), recoveries of 77% and 63%, respectively, were obtained at system flows of 275 µL min^−1^. Especially for acetonitrile, the nitrate recovery of 63% is promising, as C = N bonds are known to be recalcitrant under acidic persulfate oxidation conditions and the bond energy in triple C-N bonds is even higher [27].

## Conclusion

To further increase the oxidation rates, the concentration of oxidation agent or the residence time in the oxidation reactor has to be increased in order to apply the oxidation procedure for the measurement of isotope ratios, as almost complete conversion would be necessary for accurate CSIA. Besides an increase in oxidation agent concentration, a change of the oxidation reactor design could be necessary to increase the nitrate recovery. In the standard design, the reactor consists of a stainless-steel tubing that is coiled around a heating cartridge, and the length of this capillary could be increased to allow longer reaction times without changes in the flow of the system. Because stainless steel is not robust against vanadium(III)-chloride in concentrated HCl a glass capillary reactor has to be used (see [29]). As increased reactor volumes after the LC separation also increase the diffusion and would negatively affect the separation, the changes have to be balanced in a way that close to 100% recovery is obtained without compromising the previously obtained chromatographic separation. To integrate the wet chemical oxidation at alkaline conditions into the measurement of compound-specific nitrogen isotope ratios, the oxidation has to be combined with gas separation and reduction steps. After the oxidation to nitrate, published techniques such as the nitrate reduction to NO by V(III)Cl_3_ solution can be used to transform nitrate into a gaseous species that can be separated by the membrane system of the LC-IRMS interface [37, 38]. NO could be measured directly on the IRMS system, or further reduction to N_2_ is performed by a copper filled gas phase reduction oven, which would in addition scavenge excess oxygen produced during the initial oxidation process [36]. In this combination, LC-IRMS could become a powerful tool for the measurement of compound-specific dual isotope information of carbon and nitrogen on a single analytical platform.

By comparison of the oxidation products formed during the oxidation by heat-activated persulfate at acidic and alkaline conditions, suitable reaction conditions for the transformation to nitrate of nitrogen bound in organic analytes or present as ammonium could be identified.

The oxidation at alkaline pH conditions could successfully be transferred from a batch reactor system to the continuous oxidation in a flow‐through system of a commercially available LC‐IRMS interface. The oxidation conditions of the batch reactor experiments were successfully transferred to the oxidation reactor of the LC-IMRS interface. For different model compounds, 63 to 100% of the initially present nitrogen could be transformed to nitrate within 43 s reaction time. The reaction time in the interface was identified as the main parameter for optimization of the nitrate recovery. Further improvements of the nitrate recovery rate could be realized by increasing the length of the heated zone of the flow-through reactor system. With the discussed reaction mechanisms from NO_3_^−^ to N_x_O_y_ and final reduction to N_2_, compound-specific *δ*^15^N measurements could be realized by the addition of a second wet chemical reduction reactor followed by a combined gas phase NO to N_2_ reaction with the benefit of the simultaneous removal of excess O_2_ [36].

## Supplementary Information

Below is the link to the electronic supplementary material.

Supplementary file1 (DOCX 18 KB)
