# Supplementary material for: Alkaline persulfate oxidation as an intermediate step for the development of a wet chemical oxidation interface for compound-specific δ15N analysis by LC-IRMS
Source: Anal Bioanal Chem. 2025 Feb 22;417(10):2085–96. doi: 10.1007/s00216-025-05795-2 (PMC11961470; doi:10.1007/s00216-025-05795-2)
Supplement: Supplementary file 1 — Supplementary file1 (DOCX 18 KB) [file 216_2025_5795_MOESM1_ESM.docx]

**Supporting Information for Alkaline Persulfate Oxidation as an Intermediate Step for the Development of a Wet Chemical Oxidation Interface for Compound-Specific *δ*^15^N Analysis by LC-IRMS**

Daniel Köster ^1,5^, Tobias Hesse^1,4^, Felix Niemann^1^,Maik A. Jochmann, ^1^, Torsten C. Schmidt ^1,2^

^1^ Instrumental Analytical Chemistry, University of Duisburg-Essen, Universitätsstr. 5, 45141 Essen, Germany

^2^ University of Duisburg-Essen, Centre for Water and Environmental Research (ZWU) Universitätsstr. 5, 45141 Essen, Germany

^4^ Probenahmedienst Feststoffe, Ressourcen- und Qualitätsmanagement, Landesamt für Natur, Umwelt und Verbraucherschutz NRW, Wuhanstr. 6, 47051 Duisburg, Germany

^5^ Institut Für Arbeitsschutz der Deutschen Gesetzlichen Unfallversicherung (IFA), Alte Heerstraße111, 53757 Sankt Augustin, Germany

**Table S1** Influence of the HPLC flow rate, oxidation reactor temperature and presence of an organic analyte (156.3 mg L^-1^ ≙ 50 mg L^-1^ C) on the pH of the LC-IRMS interface effluent

|  | |  |  |  |  |  |
| --- | --- | --- | --- | --- | --- | --- |
| **Eluent flow HPLC (water)**  **(µL min^-1^)** | **NaOH**  **(50 g L^-1^)** | **Na_2_S_2_O_8_**  **(100 g L^-1^)** | **Total Flow**  **(µL min^-1^)** | **pH**  **(30 °C)** | **pH**  **(100 °C)** | **pH (100 °C + Analyte)** |
| 200 | 50 | 50 | 300 | 12.71 | 12.61 | 12.58 |
| 300 | 50 | 50 | 400 | 12.64 | 12.55 | 12.53 |
| 400 | 50 | 50 | 500 | 12.57 | 12.48 | 12.48 |
| 500 | 50 | 50 | 600 | 12.53 | 12.46 | 12.45 |
| 600 | 50 | 50 | 700 | 12.54 | 12.36 | 12.43 |
